# Supplementary material for: Protective Effects of ALDH1A Enzyme Inhibition on Helicobacter-Induced Colitis in Smad3−/− Mice are Associated with Altered α4ß7 Integrin Expression on Activated T Cells
Source: Nutrients. 2020 Sep 24;12(10):2927. doi: 10.3390/nu12102927 (PMC7599670; doi:10.3390/nu12102927)
Supplement: Supplementary file 1 [file nutrients-12-02927-s001.pdf]

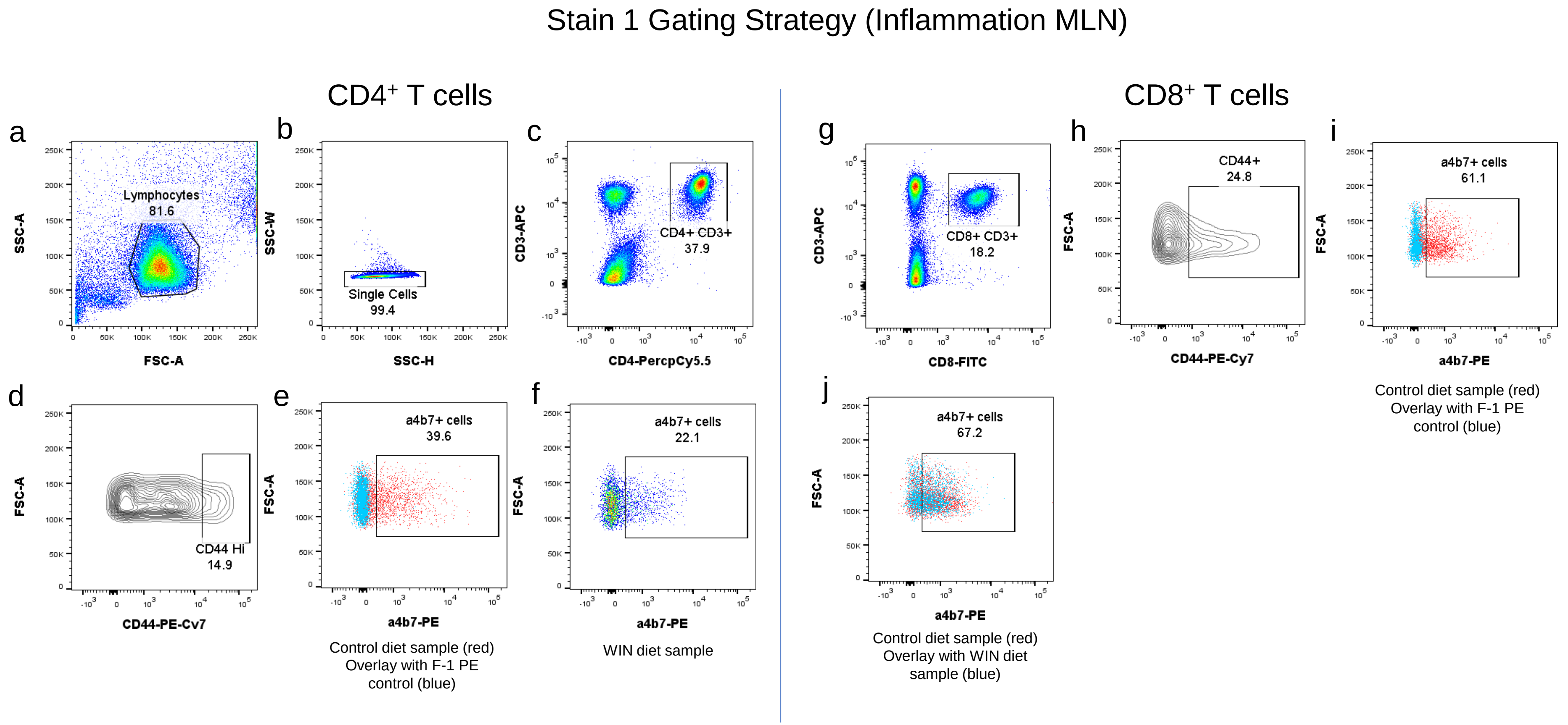

Figure S1. Gating strategies for activated $\alpha 4\beta 7^{+}$ T cells in the MLN (Stain 1). A lymphocyte gate was determined based on a forward and side scatter area plot (**a**) and doublets excluded based on side scatter height and width (**b**). Plots are from a control diet sample except where indicated. Gating sequence: lymphocytes (a) -> single cells (**b**) -> $CD3^{+}$ $CD4^{+}$ (**c**) or $CD3^{+}CD8^{+}$ (**g**) -> $CD44^{Hi}$ (for $CD4^{+}$ T cells, d) or $CD44^{+}$ (for $CD8^{+}$ T cells, h)-> $\alpha 4\beta 7^{+}$ (**e, f, i, j**). Staining of $\alpha 4\beta 7$ integrin is shown for a control sample in red in (e) and (i), compared to a fluorescence minus 1 (F-1) stain for PE (light blue), which was used to set the gate. A WIN 18,466-treated sample is shown in (**f**) and (**j**). In (**j**), the WIN 18,466-treated sample (light blue) is overlaid with a control diet sample (red). $CD4^{+}$ T cell gating strategy (**a–f**). $CD8^{+}$ T cell gating strategy (**g–h**).

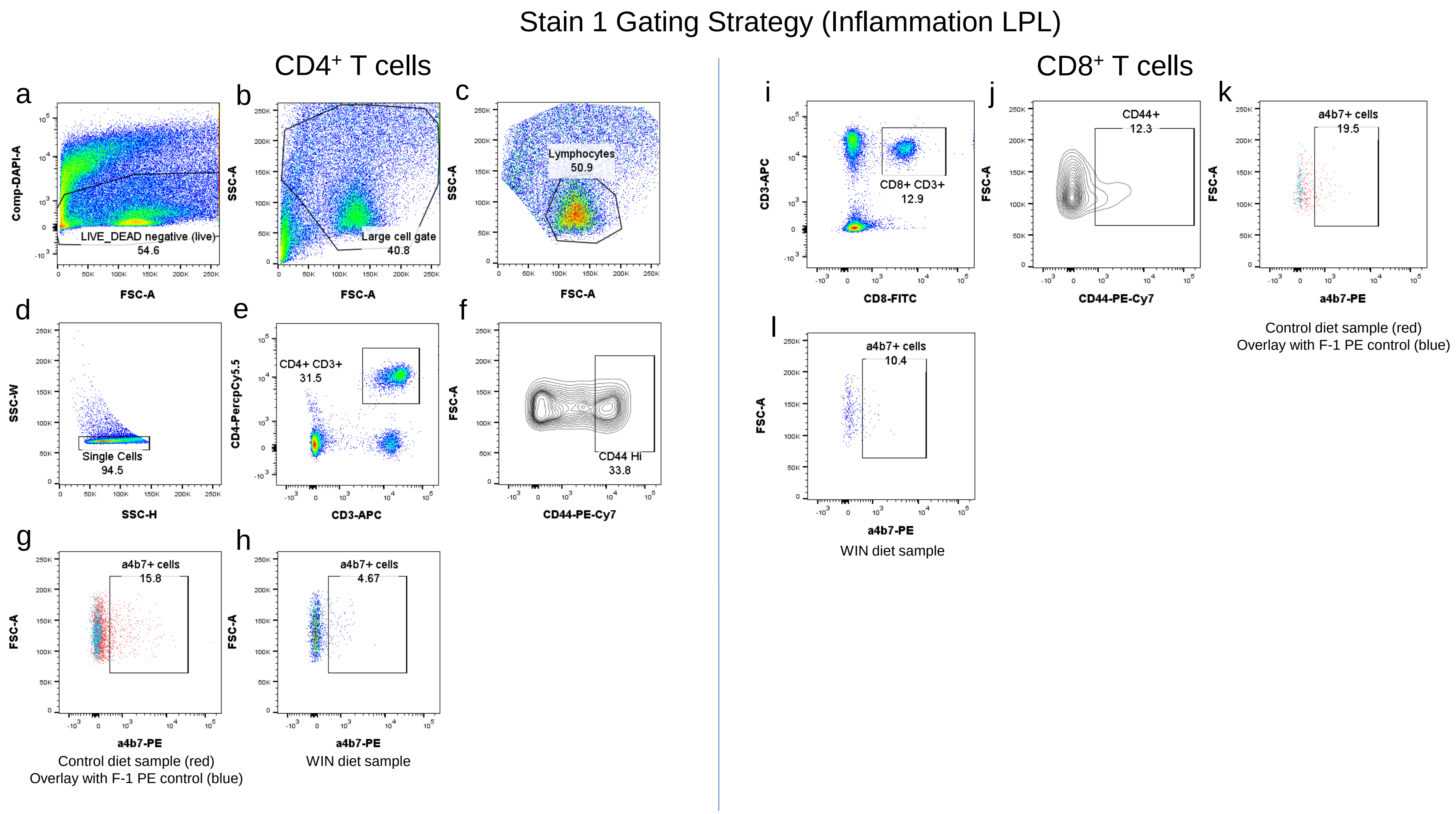

Figure S2. Gating strategies for activated α4β7⁺ T cells in LPL (Stain 1). A similar gating strategy was used as Figure S1 except that Live/Dead staining (**a**) was included for LPL samples. Plots are from a control diet sample except where indicated. The Large cell gate (**b**) was not used in this analysis except that the Lymphocyte gate (**c**) was made through the Large cell gate. A control diet sample (red) is shown overlaid with a fluorescence minus one (F-1) PE stain (light blue) in (**g**) and (**k**). A WIN 18,466-treated sample is shown in (**h**) and (**i**). CD4⁺ T cell gating strategy (**a**–**h**). CD8⁺ T cell gating strategy (**i**–**l**).

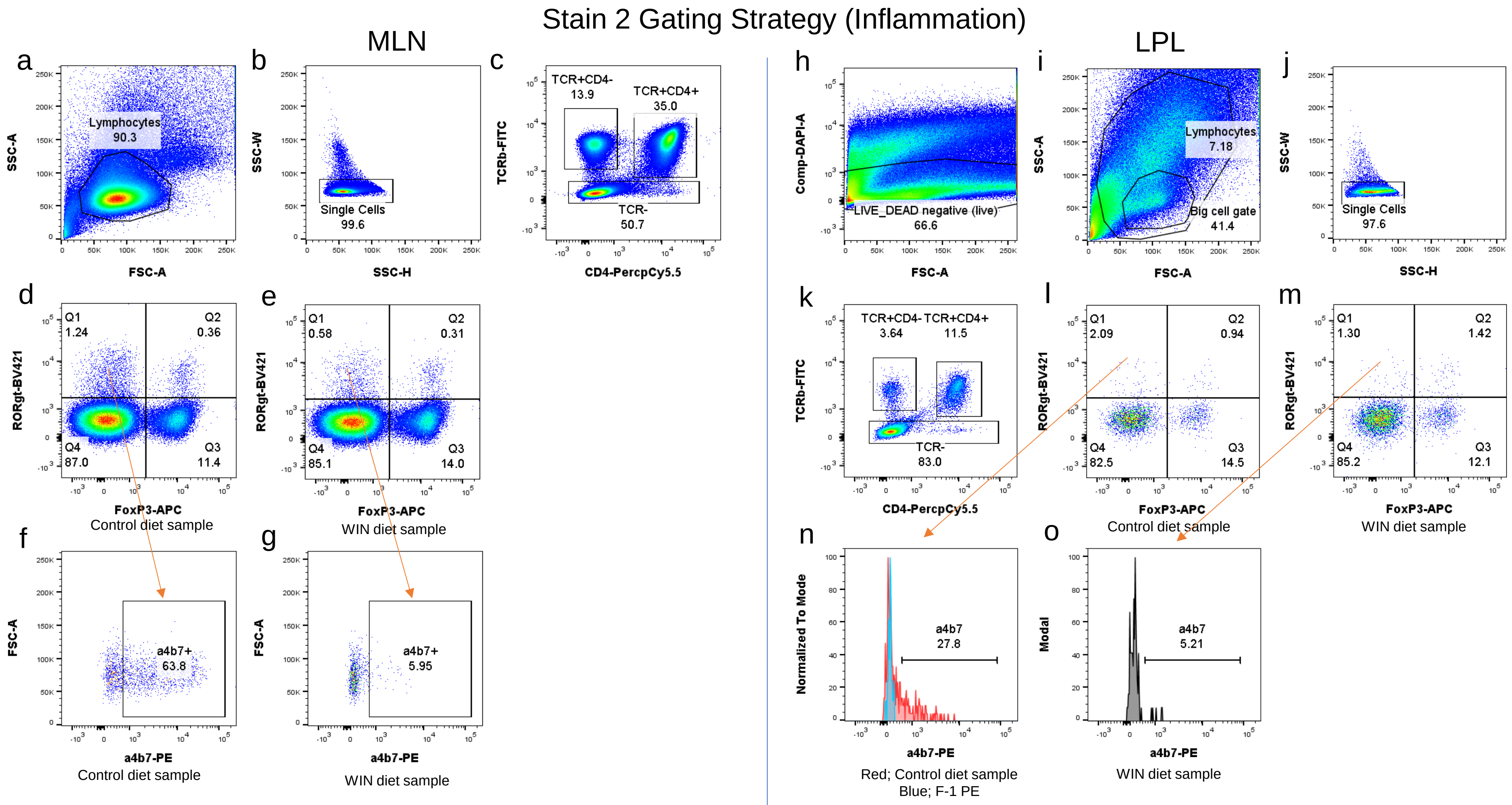

Figure S3. Gating strategies for Stain 2, Tregs (Foxp3⁺) and Th17 cells (RORγt⁺) in the inflammation experiment. Strategies are shown for MLN (**a–g**) and LPL (**h–o**). Plots are from a control diet sample except where indicated. Lymphocytes (a, i) -> single cells (b, j) -> TCR⁺ CD4⁺ (c, k) -> Quadrant gate of RORγt vs. FoxP3 (**d, e, l, m**) -> α4β7⁺ (**f, g, n, o**) of Q1 is shown (not shown for Q2). Percent α4β7⁺ of RORγt⁺FoxP3⁻CD4⁺ T cells (Q1) are shown for a control sample (**f, n**) and a WIN

18,466-treated sample (**g, o**). In (**n**), a control diet sample (red) is overlaid with a fluorescence minus 1 (F-1) sample for PE. Histograms were used in LPL for $\alpha 4\beta 7$ for better determination of the positive gate. For LPL (h-o), LIVE_DEAD staining (**h**) was also included.

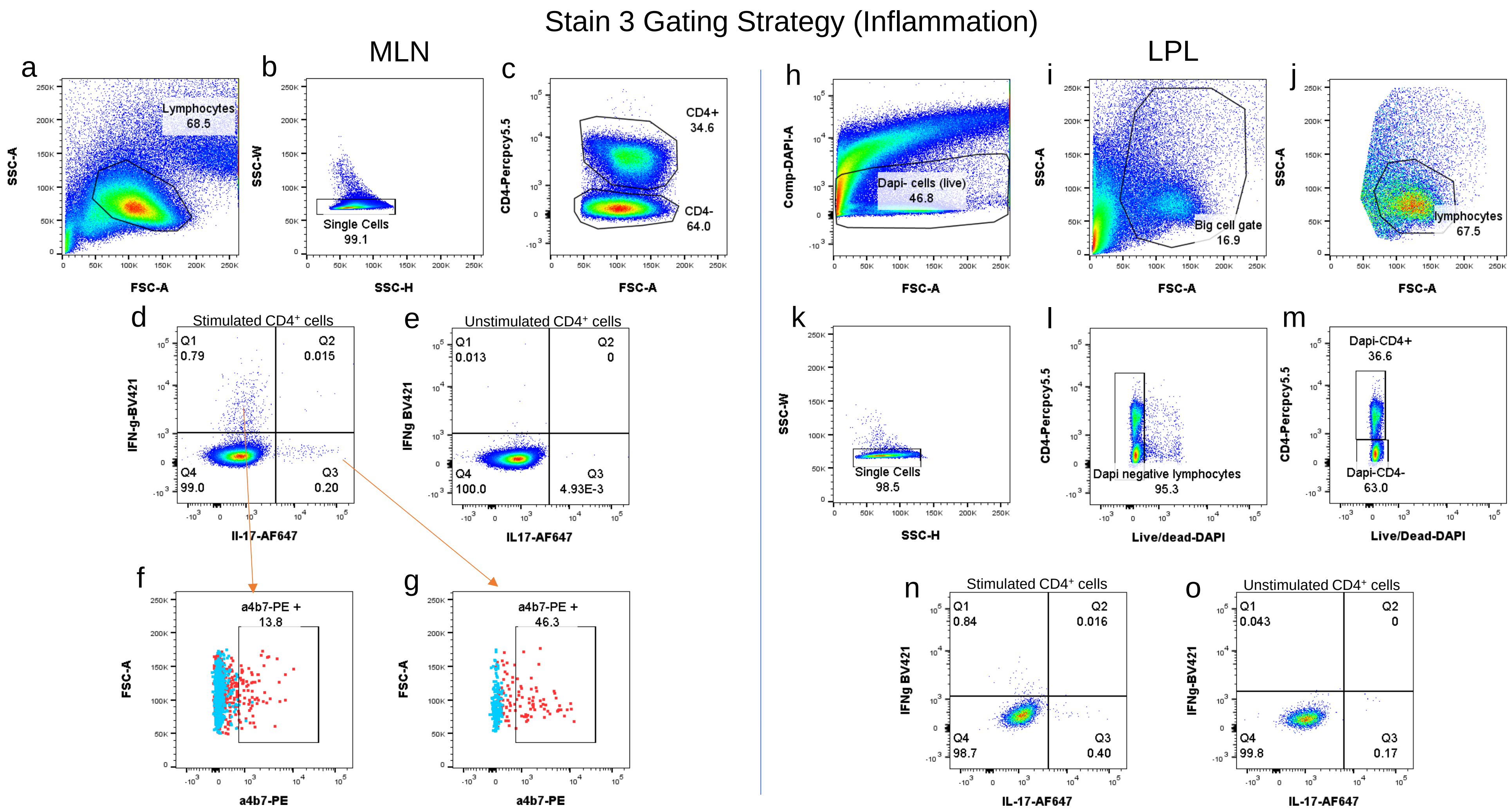

Figure S4. Gating strategies for IC cytokine stains (Stain 3). Strategies are shown for MLN (**a–g**) and LPL (**h–o**). Plots are all from a control diet sample. Gating sequence for MLN: lymphocytes (**a**) -> single cells (**b**) -> CD4$^+$ (**c**) -> Quadrant gates for IFNγ vs. IL-17 (**d**) -> $\alpha 4\beta 7^+$ (**f, g**). Gating sequence for LPL: LIVE_DEAD

negative gate (**h**) -> big cell gate (**i**) -> lymphocytes (**j**) -> single cells (**k**) -> narrower LIVE gate (**l**) -> CD4$^+$ (**m**) -> Quadrant gates for IFNγ vs. IL-17 (**n**). Cytokine quadrant gates of unstimulated samples (**e, o**) are also shown. α4β7 integrin staining is shown for IFNγ^+ stimulated CD4$^+$ cells (**f**, Red) and IL17$^+$ stimulated CD4$^+$ cells (**g**, Red). A fluorescence minus 1 (F-1) control for PE (Light Blue, **f, g**) was used to set the α4β7 gate. There were too few cytokine positive cells in LPL and therefore α4β7 was not evaluated.

Effects of RA reduction on MLN T cell phenotypes during steady state

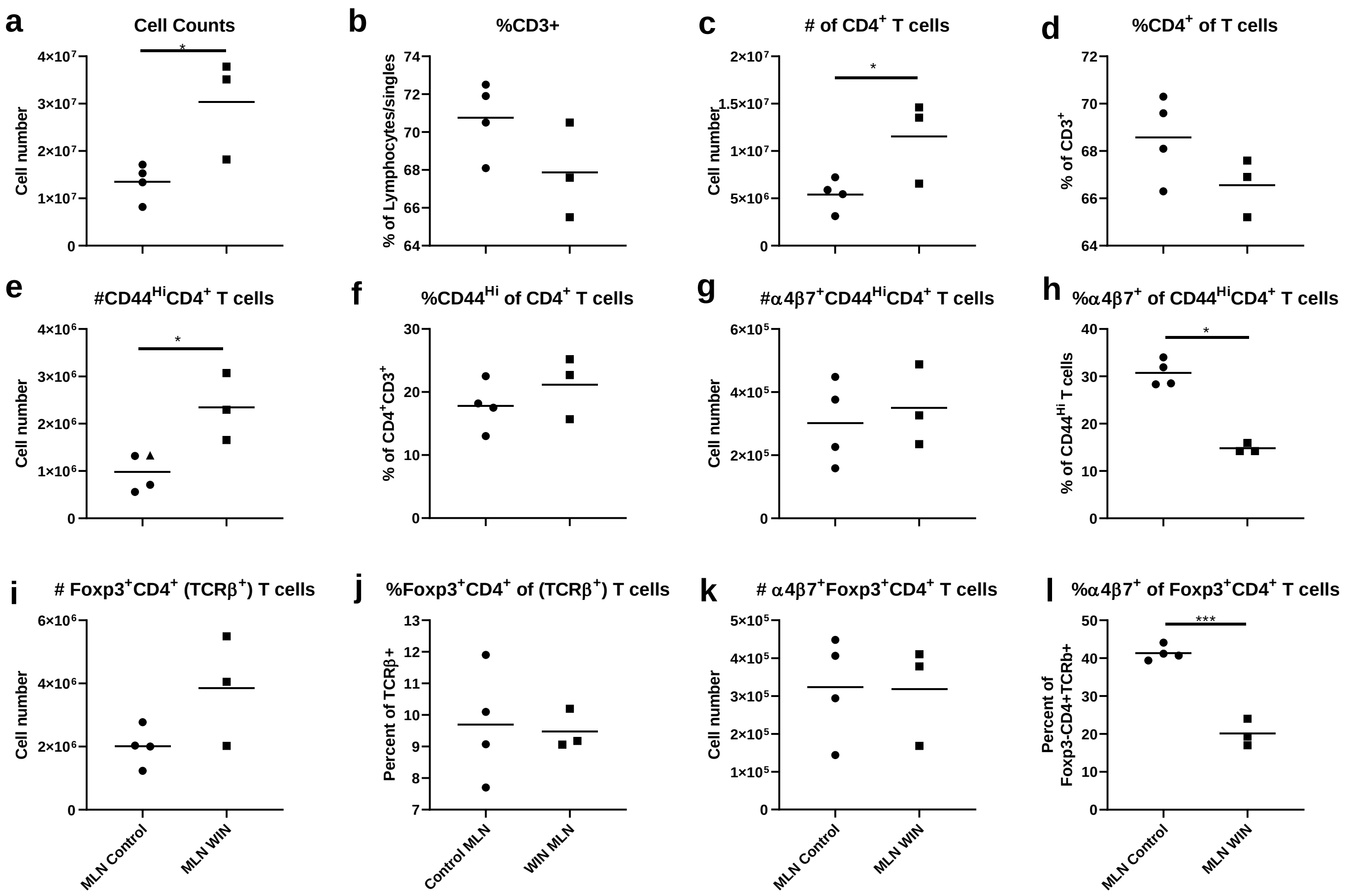

Figure S5. Effects of RA reduction on MLN T cells. Cells from MLN of mice fed WIN 18,446-containing or control diets (N = 3 and 4 mice per group, respectively) for 3 weeks were stained with antibodies in two different stains similar to those in Table 1, (**a**–**h**) Stain 1 and (**i**–**l**) Stain 2, and evaluated using flow cytometry. (**a**) Total live cell counts (hemocytometer) of MLN preparations. (**b**) Percent $CD3^+$ T cells. (**c**) Numbers of $CD4^+$ $CD3^+$ T cells. (**d**) Percent $CD4^+$ of $CD3^+$ T cells. (**e**) Numbers of activated ($CD44^{Hi}$) $CD4^+$ T cells. (**f**) Percent activated ($CD44^{Hi}$) of $CD4^+$ T cells. (**g**) Numbers of $\alpha 4\beta 7^+$ activated $CD4^+$ T cells. (**h**) Percent $\alpha 4\beta 7^+$ of activated

CD4$^+$ T cells. (**i**) Numbers of Foxp3$^+$ CD4$^+$ T (TCRβ$^+$) cells. (**j**) Percent Foxp3$^+$ CD4$^+$ of T (TCRß$^+$) cells. (**k**) Numbers of α4β7$^+$Foxp3$^+$ CD4$^+$ T cells. (l) Percent α4β7$^+$ of Foxp3$^+$ CD4$^+$ T cells. *$p < 0.05$, **$p < 0.01$, ***$p < 0.001$ pair-wise comparisons via student's T Test or Mann-Whitney test. Note the different origin of y-axes in b, d, and j. Data shown are from one of two independent experiments. The other one is shown in Figure 2.

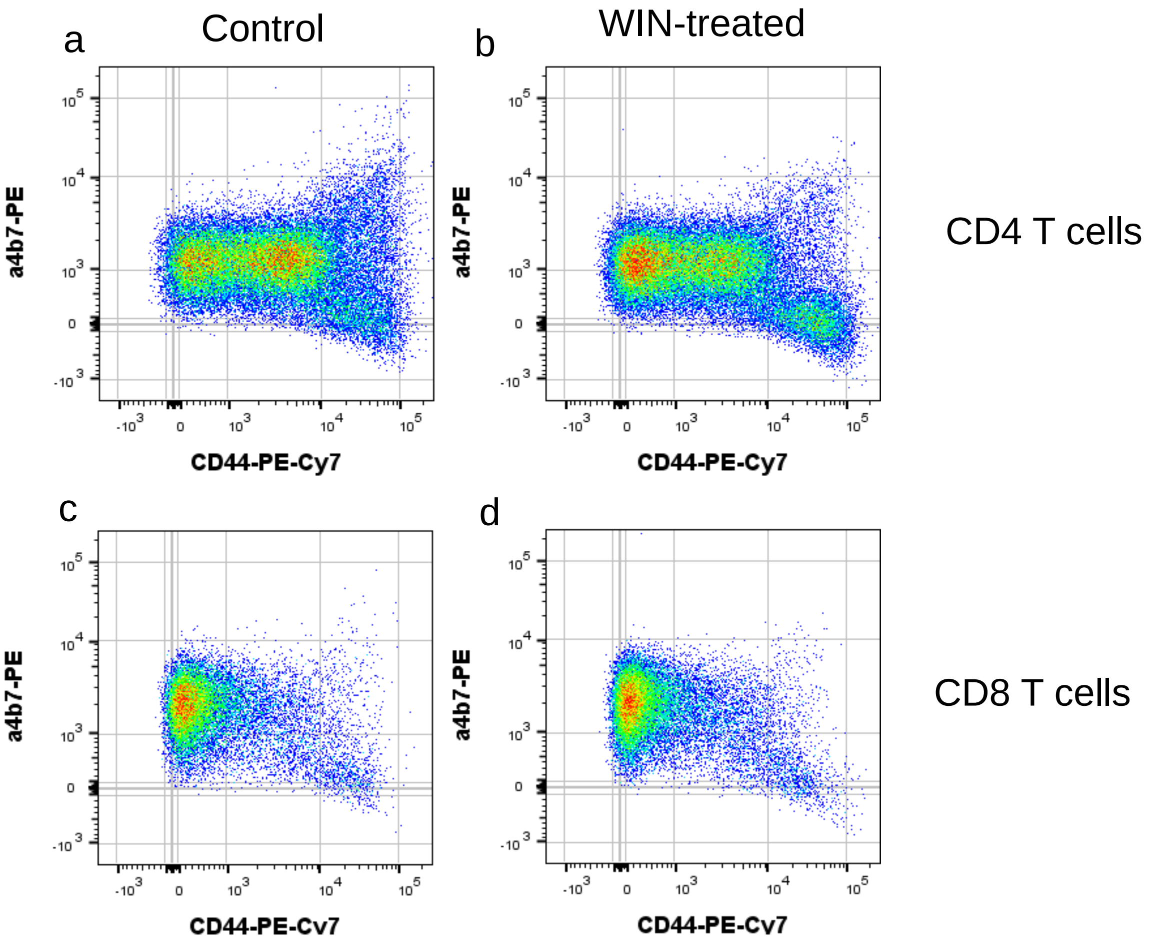

Figure S6. CD44 vs. α4β7 integrin staining in steady state MLN T cells from control diet vs. WIN 18,466-treated mice. Cells are gated through CD4$^+$ (**a, b**) or CD8$^+$ (**c, d**) T cells. The noticeable decrease in α4β7 $^+$CD4$^+$CD44^{Hi} cells with WIN 18,466-treatment compared to control diet treatment (**a** vs. **b**) is not observed in CD8$^+$ CD44$^+$ T cells (**c** vs. **d**).

Complementary Data to Figure 3

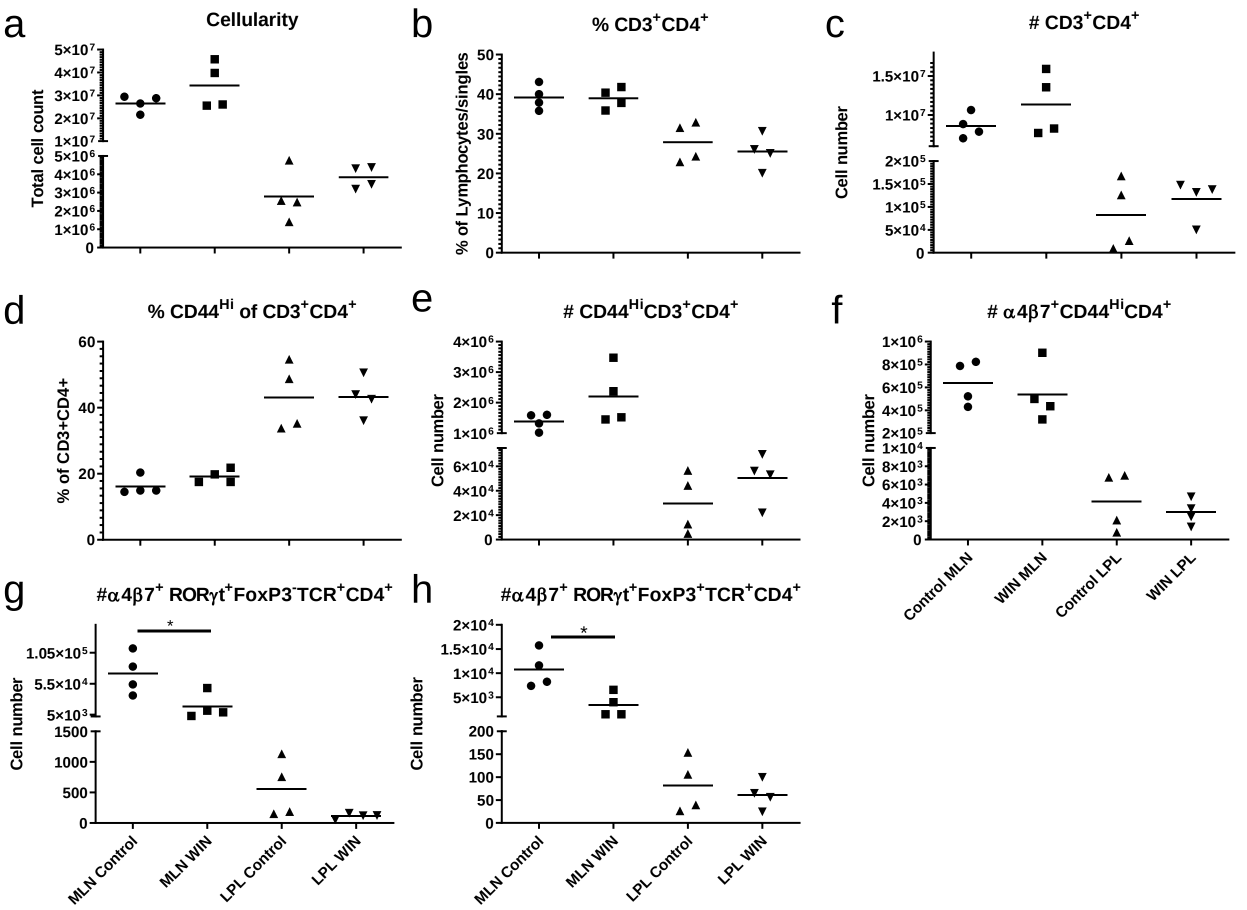

Figure S7. Supplementary data complementing Figure 3 of the inflammation T cell subsets experiment. (**a**) Cellularity of MLN and LPL samples. (**b, d**) Percentages and (**c, e**) cell numbers for (**b, c**) $CD4^+$ T cells, (**d, e**) activated $CD4^+$ T cells and (**f**) numbers for $\alpha4\beta7^+$ activated cells and (**g**) effector $Foxp3^-$ and (**h**) regulatory $Foxp3^+$ $CD4^+$ T cells. Split axes are used in graphs of cell numbers due to the large difference in cell numbers harvested from MLN vs. LPL. *$p < 0.05$.
